# Mapping microglial mechanisms in Alzheimer’s disease: a comprehensive analysis

**DOI:** 10.3389/ebm.2025.10808

**Published:** 2025-12-03

**Authors:** Xiaofang Wang, Yuqing Guo, Yonghan Zha, Shuling Wang, Weihua Yang, Qianfang Jia

**Affiliations:** 1 Hebei University of Chinese Medicine, Shijiazhuang, China; 2 Department of Traditional Chinese Medicine, Affiliated Children’s Hospital of Jiangnan University, Wuxi Children’s Hospital, Wuxi, China; 3 The First Clinical College of Henan Medical University, Xinxiang, China; 4 Shenzhen Eye Hospital, Shenzhen Eye Medical Center, Southern Medical University, Shenzhen, China; 5 Department of Children’s Rehabilitation, First Affiliated Hospital of Xinxiang Medical University, Xinxiang, China

**Keywords:** microglia, Alzheimer’s disease, mechanism, hotspot, trend

## Abstract

Microglia, the brain’s primary immune cells, play crucial roles in Alzheimer’s disease (AD) pathogenesis. However, existing research remains abundant yet fragmented. Therefore, this study aimed to systematically identify hotspots and trends in microglia-related AD research, while providing an in-depth analysis of the underlying mechanisms to advance mechanistic understanding and therapeutic development. To achieve this, articles on microglia in AD were retrieved from the Web of Science Core Collection (WoSCC) database, and bibliometric analysis was performed using the WoSCC platform and CiteSpace 6.3.R1, with a focus on global collaboration, institutional and journal contributions, keyword bursts, and high-impact articles to comprehensively elucidate the underlying mechanisms. In total, 1,043 articles from 67 countries and regions were included.Among them, the United States led with 484 articles and an H-index of 100, followed by China with 276 articles. The University of California system (77 articles) and Harvard University (74 articles) had the highest H-index, both at 41. Journal of Neuroinflammation published the most articles (57 articles). Burst keywords persisting until 2024 included “memory,” “NLRP3 inflammasome,” and “system.” High-impact studies emphasized microglial roles in AD pathology, including Aβ clearance, synaptic pruning, inflammation, metabolism, phenotype shifts, immune memory, and genetic variation. Overall, microglial mechanisms are at the forefront of AD research. The United States leads in both article number and influence, followed by China. The University of California system and Harvard University demonstrate the greatest output and impact. *Journal of Neuroinflammation* is the leading journal. Microglial NLRP3 activation, system-level interactions, and memory impairment have emerged as key research hotspots in AD. Future research will focus on microglial mechanisms and therapeutic targets in AD.

## Impact statement

Alzheimer’s disease (AD) remains one of the most pressing challenges in aging research, with microglia increasingly recognized as central players in disease onset and progression. Despite abundant publications, existing studies are fragmented and lack a systematic overview of global trends and emerging directions. This work fills that gap by providing the first comprehensive bibliometric mapping of microglial research in AD over the past decade. It highlights how microglia contribute to aging-related processes, including inflammation, metabolic shifts, immune memory, and genetic susceptibility, and identifies the key institutions, journals, and research themes driving the field. By integrating this information, the study offers a clearer picture of how microglial biology intersects with aging mechanisms in AD. This perspective not only advances understanding of disease pathology but also helps guide future research toward innovative therapeutic strategies targeting microglial dysfunction in aging-related neurodegeneration.

## Introduction

Alzheimer’s disease (AD) is a prototypical neurodegenerative disorder, characterized by amyloid-β (Aβ) plaque deposition and abnormal tau phosphorylation [1]. Increasing evidence indicates that AD is accompanied by disrupted neuroimmune homeostasis, with neuroinflammation playing a central role in its pathogenesis [2, 3]. Microglia, the principal immune cells of the central nervous system (CNS), display functional abnormalities in AD. These abnormalities not only sustain chronic neuroinflammation but also impair Aβ clearance, synaptic remodeling, and neuronal survival, thereby positioning microglia as both initiators and amplifiers of AD pathology [4–6].

Mechanistically, neuronal overexpression of cathepsin S activates the CX3CL1–CX3CR1 axis and JAK2–STAT3 signaling, driving microglia toward a pro-inflammatory M1 phenotype and disrupting lysosomal protease balance, which intensifies neuroinflammation and accelerates disease progression [7]. Activated microglia further secrete pro-inflammatory cytokines via the MAPK, PI3K, and JAK/STAT pathways, establishing a positive feedback loop with peripheral immune cells that exacerbates neuronal injury [8]. In addition, microglial upregulation of the complement system under AD conditions induces aberrant phag [9]. Dysregulation of the TREM2/PGRN signaling axis further diminishes microglial capacity to clear Aβ plaques, while TREM2-driven microglial activation independently aggravates synaptic injury [10]. Collectively, these findings highlight microglia as a pathological hub linking Aβ deposition, tau abnormalities, synaptic dysfunction, and neuroinflammation in AD.

Given their central role, microglia have emerged as promising therapeutic targets in AD, with novel strategies including TREM2 agonists [11, 12], anti-inflammatory modulation [13, 14], and gene-editing approaches [15, 16]. However, despite rapid advances, microglia-related AD research remains highly heterogeneous, encompassing diverse mechanisms and intervention strategies. This fragmentation underscores the need for an integrative perspective to clarify research hotspots, developmental trajectories, and translational potential. To this end, the present study performed a bibliometric analysis of publications from the past decade indexed in the Web of Science Core Collection (WoSCC), aiming to map the evolving landscape of microglia-related AD research and to conduct an in-depth analysis of the underlying mechanisms, thereby providing systematic insights that may inform the development of novel therapeutic interventions and their clinical translation.

## Materials and methods

The WoSCC serves as a standard dataset for journal impact and institutional performance indicators and has become a key resource in bibliometric research [17, 18]. To systematically identify the related literature, we established a stringent selection strategy. In the WoSCC database, we set the topic as “microglia*” and the additional topic as “Alzheimer*” or “AD”, restricting the language to English, the document type to“article,” and the publication date range from January 1, 2015 to December 31, 2024, resulting in 2,856 retrieved articles.

**FIGURE 1:**
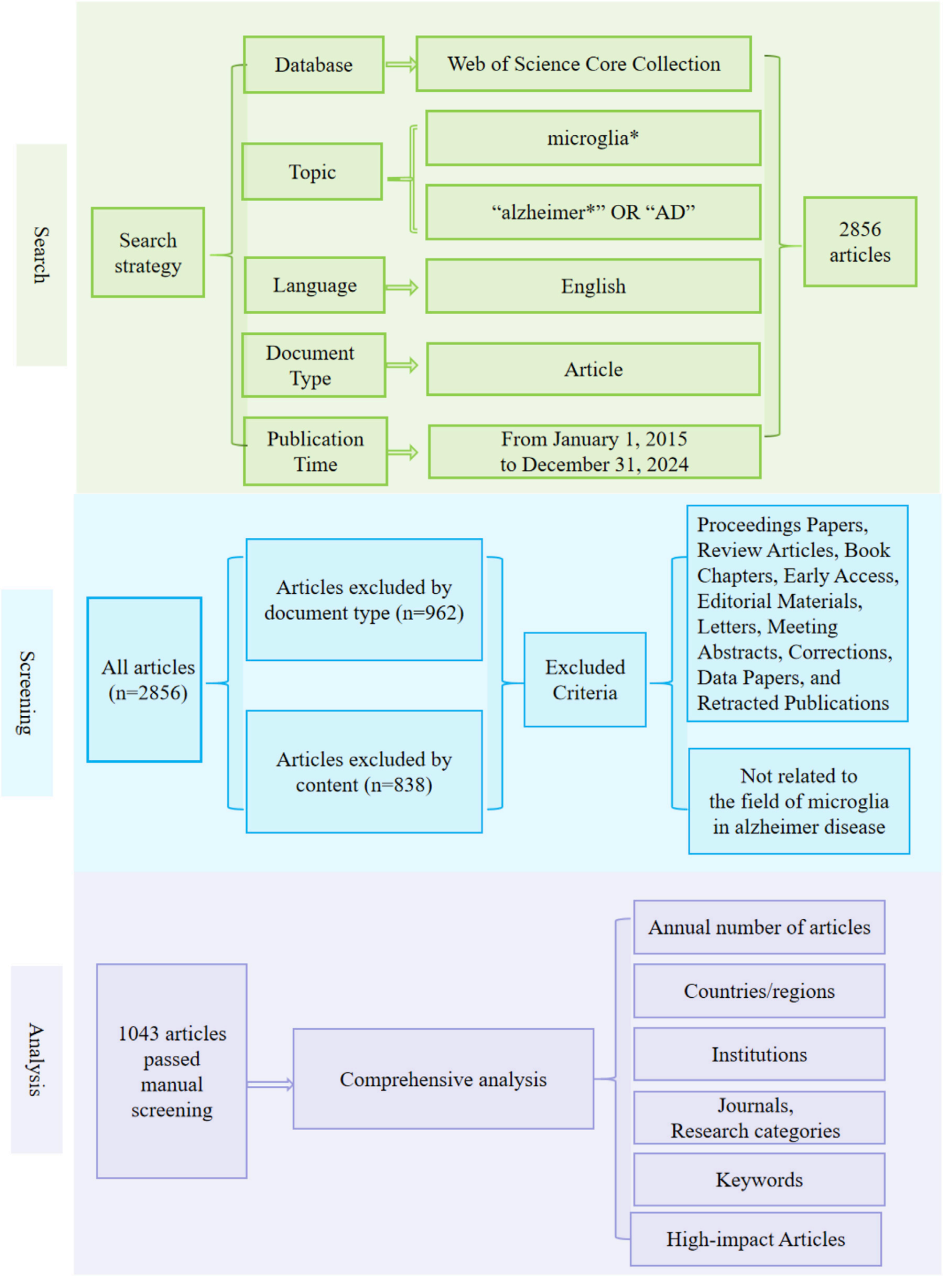
Frame flow diagram showing the detailed selection criteria and analysis steps for the study of microglia in AD.

All retrieved records were imported into CiteSpace 6.3.R1 software for data processing. Duplicate entries were automatically detected and removed based on DOI, title, author, and publication year. Furthermore, a manual screening procedure was conducted to ensure the quality of the dataset, and the following criteria were applied: (1) documents including conference proceedings, review articles, book chapters, early access, editorial materials, letters, meeting abstracts, corrections, data papers, and retracted publications were excluded by document type; (2) articles were then filtered based on their content to ensure alignment with the target topic—for instance, studies addressing only AD or only microglia were excluded. During the manual screening, a double-blind review mechanism was implemented. Two independent reviewers (XW and YG) screened the records according to the above criteria without access to each other’s decisions. Any discrepancies were resolved through discussion or by consultation with a third-party expert adjudicator (QJ). As a result, a total of 1,043 high-quality articles were ultimately included following manual screening. Moreover, CiteSpace was employed for collaboration network analysis (by country/region, institution, and journal) and for burst keywords identification, and the WoSCC analysis system was used to analyze annual and regional article counts. The full literature screening process is illustrated in Figure 1. In addition, high-impact articles were subjected to in-depth analysis to better showcase pivotal findings in the field of microglia in AD.

## Results

### Annual number of articles

**FIGURE 2:**
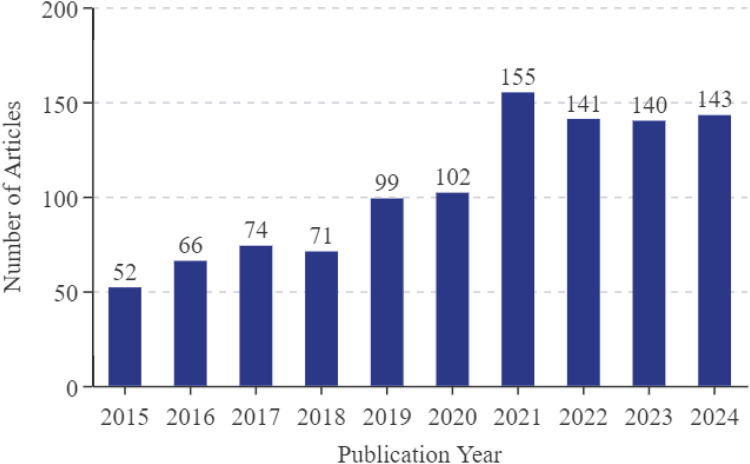
Annual number of articles on microglia in AD.

Over the past decade, annual articles in the field of microglia in AD have consistently exceeded 50, exhibiting a general increasing trend. Between 2015 and 2020, the number of annual articles rose steadily, from 52 to 102. Beginning in 2021, the number of articles in this field has grown markedly, with an average of more than 140 articles per year from 2021 to 2024. Figure 2 depicts the annual number of articles in this field over the last decade.

### Countries or regions

**FIGURE 3:**
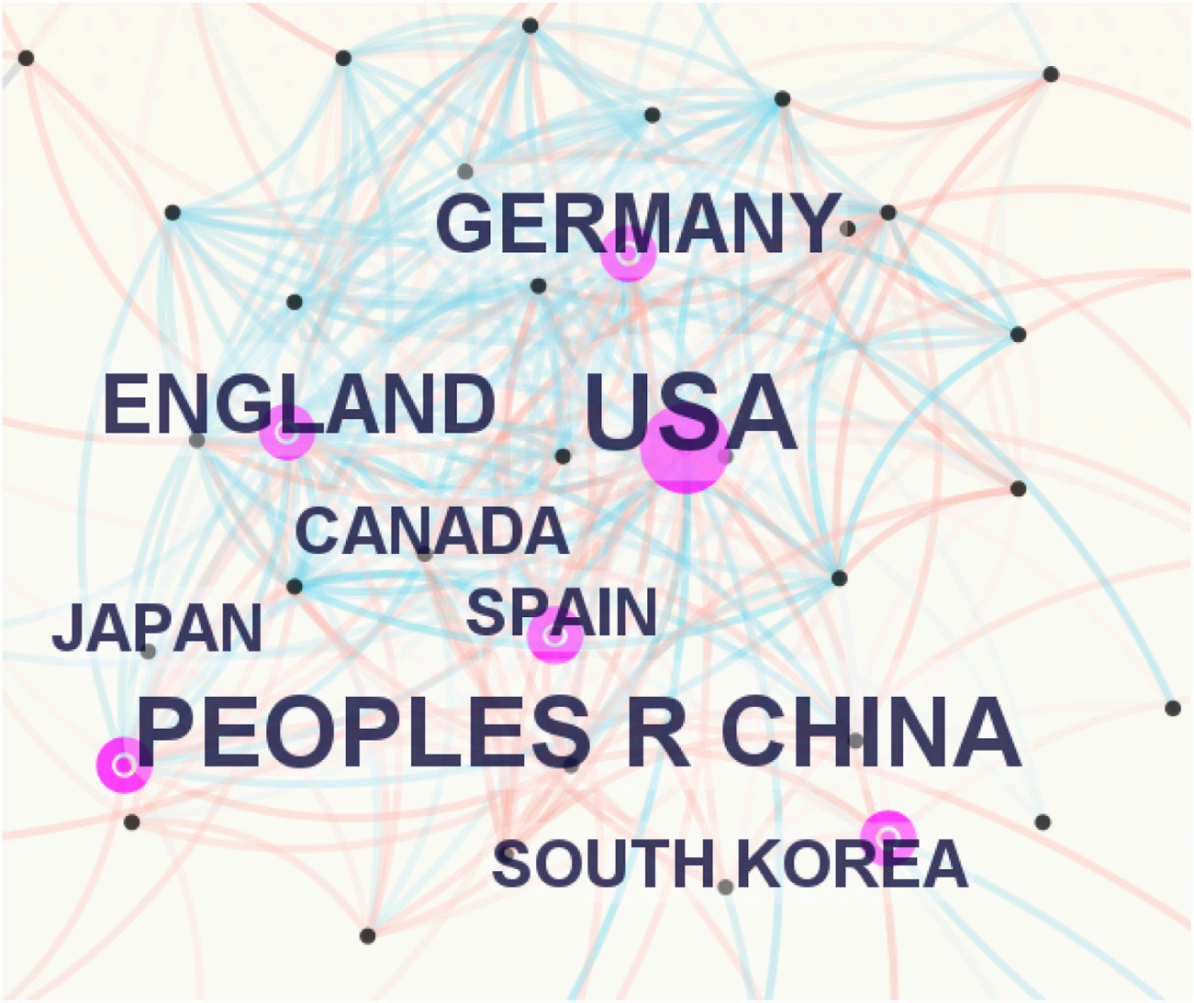
Cooperation of countries or regions that contributed to articles on microglia in AD.

These articles originated from 67 countries and regions. Figure 3 illustrates the collaboration network of countries and regions constructed with the default parameters in CiteSpace. The size of each label and node in Figure 3 is proportional to the number of articles. The largest nodes and labels belonged to the United States (484 articles), China (276), and England (112), indicating their dominant contributions. Connections between nodes indicate collaboration between countries, with more links signifying closer cooperation. Table 1 lists the top 10 countries or regions by number of articles, along with centrality scores (reflecting collaboration intensity) and H-indices (measuring academic impact). The United States ranked first with the highest H-index (100) and also demonstrated the greatest centrality (0.60). China followed with an H-index of 58 but exhibited a lower centrality score of 0.09.

**TABLE 1:** Top 10 countries or regions with articles on microglia in AD.

Rank	Countries or regions	Counts	Centrality	H-index
1	United States	484	0.60	100
2	China	276	0.09	58
3	England	112	0.14	47
4	Germany	112	0.18	53
5	Canada	54	0.02	31
6	Spain	53	0.13	26
7	Japan	53	0.00	29
8	South Korea	46	0.07	22
9	Sweden	42	0.07	21
10	Italy	41	0.13	23

### Institutions

**FIGURE 4:**
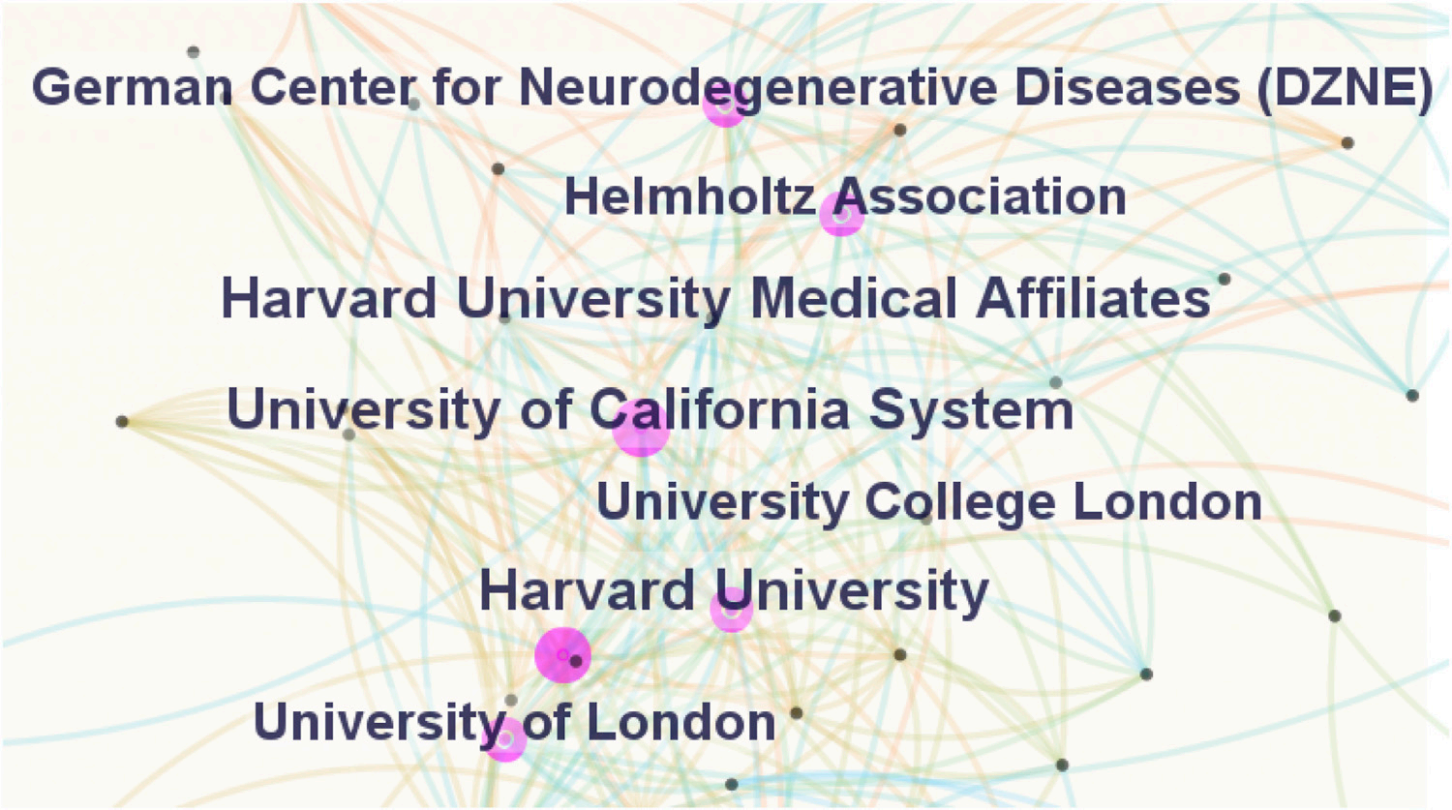
Cooperation of institutions that contributed to articles on microglia in AD.

Figure 4 presents the institutional collaboration network constructed with CiteSpace’s default settings. The size of nodes and labels of each institution in the figure is proportional to its article output, with connections representing collaborations. Table 2 lists details of the top 10 institutions based on article counts. Regarding article output, the University of California System (77 articles), Harvard University (74 articles), and Helmholtz Association (55 articles) hold the top 3 positions. The University of California System and Harvard University shared the highest H-index of 41. Of the top 10 institutions, five are based in the United States, three in Germany, and two in England.

**TABLE 2:** Top 10 Institutions with articles on microglia in AD.

Rank	Institution	Country or regions	Counts	H-index
1	University of California System	United States	77	41
2	Harvard University	United States	74	41
3	Helmholtz Association	Germany	55	35
4	Harvard University Medical Affiliates	United States	55	33
5	University of London	England	52	27
6	German Center for Neurodegenerative Diseases (DZNE)	Germany	48	33
7	University College London	England	45	25
8	Harvard Medical School	United States	41	25
9	Washington University (WUSTL)	United States	37	29
10	University of Munich	Germany	30	24

### Journals

Table 3 presents the top 10 journals ranked by article number in the research field of microglia in AD. The journals with the highest article counts were *Journal of Neuroinflammation, Journal of Alzheimer’s Disease,* and *Glia*, with 57, 41, and 31 articles, respectively, primarily covering Immunology, Neurosciences, and Multidisciplinary Sciences. Among these 10 journals, Molecular Neurodegeneration had the greatest impact, with an impact factor of 14.9.

**TABLE 3:** Top 10 journals with articles on microglia in AD.

Rank	Citing journals	Research categories	Counts	Journal impact factor 2023
1	*Journal of Neuroinflammation*	Immunology; neurosciences	57	9.0
2	*Journal of Alzheimer’s Disease*	Neurosciences	41	3.4
3	*Glia*	Neurosciences	31	5.4
4	*Nature Communications*	Multidisciplinary sciences	30	14.7
5	*International Journal of Molecular Sciences*	Biochemistry and molecular biology; chemistry, multidisciplinary	29	4.9
6	*Molecular Neurodegeneration*	Neurosciences	27	14.9
7	*Brain Behavior and Immunity*	Immunology; neurosciences; psychiatry	23	8.8
8	*Acta Neuropathologica Communications*	Neurosciences	20	6.2
9	*Molecular Neurobiology*	Neurosciences	19	4.6
10	*Neurobiology of Aging*	Geriatrics and gerontology; neurosciences	19	3.7

### Keywords

**FIGURE 5:**
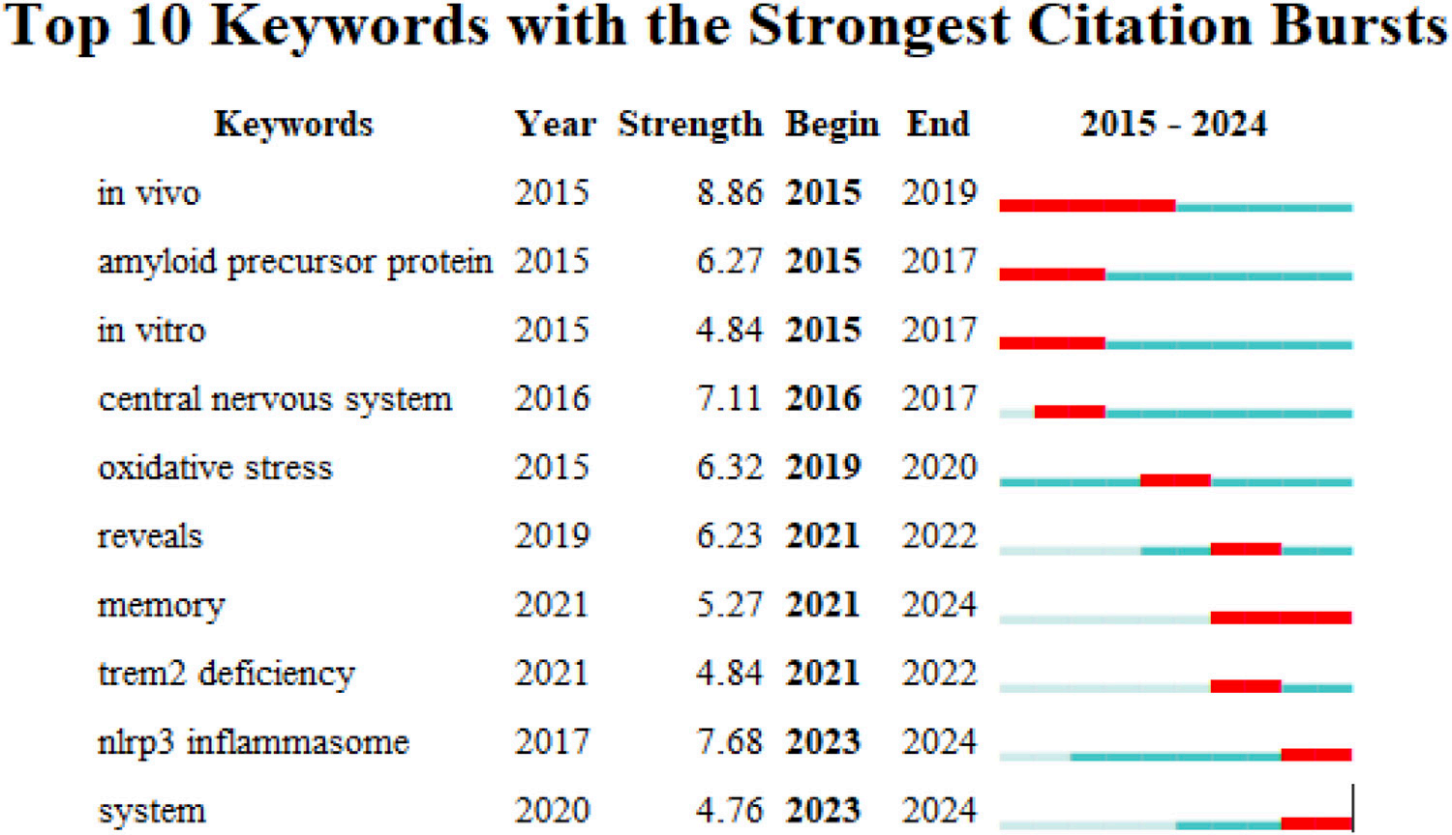
Keywords with the strongest citation bursts for publications on microglia in AD.

CiteSpace was employed to analyze co-occurring collaborative networks of keywords using the parameters: “Year Per Slice” = 1, “Top N%” = 10.0%, and “Minimum Duration” = 1. Figure 5 highlights the top 10 keywords with the strongest citation bursts, where strength indicates the intensity of the keyword’s emergence. The red squares denote the timeline of keyword surges. The keywords that persisted until 2024 include “memory,” “NLRP3 inflammasome,” and “system.”

### High-impact publications

Articles with high citation counts represent highly acknowledged research in the field of microglial research in AD and are classified as high-impact articles. Table 4 presents the top 10 articles with the highest citations in this research area. These studies focused on investigating the pathological mechanisms of AD and the functions of microglia, including induction of neurotoxic astrocytes (A1 type) via secretion of inflammatory factors, identification of novel disease-associated microglia (DAM) and their role in Aβ clearance, synaptic pruning mediated by the complement pathway, and regulation of microglial phenotype transformation by the TREM2-APOE pathway. Additionally, microglial metabolic dysfunction, epigenetic reprogramming, and inflammasome involvement, along with the effects of genetic variants on microglial functions, were elucidated.

**TABLE 4:** Articles with top 10 citation on microglia in AD.

Rank	Title of article	DOI	Times cited	Interpretation of the findings
1	Neurotoxic reactive astrocytes are induced by activated microglia	10.1038/nature21029	5043	Microglia promote A1-type astrocyte generation via IL-1α, TNF, and C1q. These astrocytes lose neuroprotective function, induce neuronal and oligodendrocyte death, and contribute to multiple neurodegenerative diseases. Blocking A1 formation protects neurons
2	A unique microglia type associated with restricting development of Alzheimer’s disease	10.1016/j.cell.2017.05.018	3198	Single-cell transcriptomics revealed disease-associated microglia (DAM). They clear Aβ through a TREM2-dependent two-step activation: Downregulation of homeostatic markers, followed by upregulation of phagocytosis-related genes
3	Complement and microglia mediate early synapse loss in Alzheimer mouse models	10.1126/science.aad8373	2134	C1q attaches to synapses before plaques and triggers microglial CR3-mediated phagocytosis. Blocking complement prevents synaptic injury, showing aberrant complement-driven pruning as a key early AD mechanism
4	The TREM2-APOE pathway drives the transcriptional phenotype of dysfunctional microglia in neurodegenerative diseases	10.1016/j.immuni.2017.08.008	1753	The TREM2-APOE pathway regulates microglial phenotypic shift from homeostasis to neurodegeneration after phagocytosis of apoptotic neurons. Therapeutic targeting restores homeostasis and protects neurons
5	TREM2 Maintains microglial metabolic fitness in Alzheimer’s disease	10.1016/j.cell.2017.07.023	805	In AD patients and TREM2-deficient mice, microglia showed defective ATP synthesis and autophagosome accumulation. Dectin-1 activation or creatine supplementation rescued metabolism and reduced neuronal injury
6	Microglia-derived ASC specks cross-seed amyloid-β in Alzheimer’s disease	10.1038/nature25158	691	Inflammasome-activated microglia release ASC specks that bind Aβ, promoting aggregation and spread. Blocking ASC suppresses pathology, linking neuroinflammation to Aβ propagation
7	Rare coding variants in PLCG2, ABI3, and TREM2 implicate microglial-mediated innate immunity in Alzheimer’s disease	10.1038/ng.3916	657	Genetic study (85,133 participants) identified AD-associated variants: PLCG2-Pro522Arg (protective), ABI3-Ser209Phe and TREM2-Arg62His (risk). These genes form a microglia-centered immune network in AD pathogenesis
8	Human and mouse single-nucleus transcriptomics reveal TREM2-dependent and TREM2-independent cellular responses in Alzheimer’s disease	10.1038/s41591-019-0695-9	651	Single-nucleus RNA sequencing revealed both TREM2-dependent microglial activation and unique human-specific responses (oligodendrocyte myelination defects, astrocyte metabolic dysregulation). TREM2 mutations weaken microglial responses
9	Innate immune memory in the brain shapes neurological disease hallmarks	10.1038/s41586-018-0023-4	630	Peripheral inflammation induces long-lasting microglial immune memory (training or tolerance). Training worsens Aβ pathology; tolerance mitigates it. Epigenetic reprogramming underlies this regulation
10	TREM2 binds to apolipoproteins, including APOE and CLU/APOJ, and thereby facilitates uptake of amyloid-beta by microglia	10.1016/j.neuron.2016.06.015	629	TREM2 mediates microglial clearance of lipoprotein–Aβ complexes. AD-associated TREM2 mutations impair this function, linking APOE-CLU-TREM2 risk network to impaired Aβ clearance.of the TREM2-APOE-CLU genetic risk network, and offering new targets for immunometabolic therapy in AD.

## Discussion

### Overall data

AD is a neurodegenerative disease characterized by β-amyloid (Aβ) accumulation and abnormal phosphorylation of tau protein [6]. Moreover, Neuroinflammation has gradually been acknowledged as a pathological factor in AD progression [19, 20]. Microglia, as the central immune regulators in the CNS, undergo dysfunction and abnormal activation, thereby triggering chronic neuroinflammation and exacerbating cognitive impairment through multisystem synaptic damage [21]. The neuroimmune mechanisms mediated by microglia have emerged as a crucial focus in AD research, with notable advancements. This study analyzed articles from the WoSCC database during the last decade to uncover research hotspots and trends related to microglia in AD. Results showed that research on microglia in AD has increased rapidly over the past decade, with expanding global participation and closer international collaboration. The United States and China remain leading contributors, and high-impact studies are concentrated in top neuroscience institutions such as the University of California system and Harvard University, as well as in specialized journals such as *Journal of Neuroinflammation*.

### Acknowledged research findings

High-impact articles have highlighted acknowledged findings regarding microglia in AD, emphasizing their pivotal role in disease pathogenesis. These articles illustrate alterations in microglial function across diverse pathological mechanisms, including Aβ clearance, synaptic pruning, inflammatory response, metabolic regulation, phenotypic transformation, immune memory, and genetic variation.

The pathological effects of microglia in AD involve multiple dimensions: (1) Impaired phagocytic and clearance capacity: Mutations in TREM2 reduce the ability of microglia to phagocytose and eliminate Aβ, resulting in increased Aβ deposition and accelerating pathological progression [22]. Furthermore, inflammasome-activated microglia release ASC specks that bind to Aβ, promoting its aggregation and spread, thereby intensifying amyloid pathology [23]. (2) Synaptic injury and dysregulated pruning: excessive activation of the complement system leads to over-pruning of synapses. C1q binds synapses prior to plaque formation, initiating synaptic pruning via microglial CR3 receptors, which worsens synaptic dysfunction [24]. (3) Neuroinflammation and glial cytotoxicity: microglia release inflammatory factors such as IL-1α, TNF, and C1q, driving the formation of neurotoxic A1 astrocytes that cause neuronal and oligodendroglial loss [25].

Microglia also exhibit intrinsic pathological changes: (1) Disrupted metabolism: under TREM2 deficiency, microglia accumulate autophagosomes and show impaired ATP production due to mitochondrial dysfunction. TREM2 supports microglial function by regulating energy metabolism [26]. (2) Dysregulated phenotypic switching: the TREM2–APOE axis orchestrates the transition from homeostatic to disease-associated microglial phenotypes (e.g., DAM). Mutations disrupt this process and contribute to neurodegeneration [27, 28]. (3) Immune imprinting and epigenetic remodeling: peripheral inflammation triggers microglia to develop prolonged immune memory. While trained immunity exacerbates pathology, immune tolerance mitigates it. Furthermore, epigenetic reprogramming is critically involved in the neuropathology of AD [29]. (4) Genetic influences: AD-associated genetic variants (e.g., TREM2 mutations) impair microglial Aβ clearance and neuroprotection, further worsening disease progression [26, 30, 31].

**FIGURE 6:**
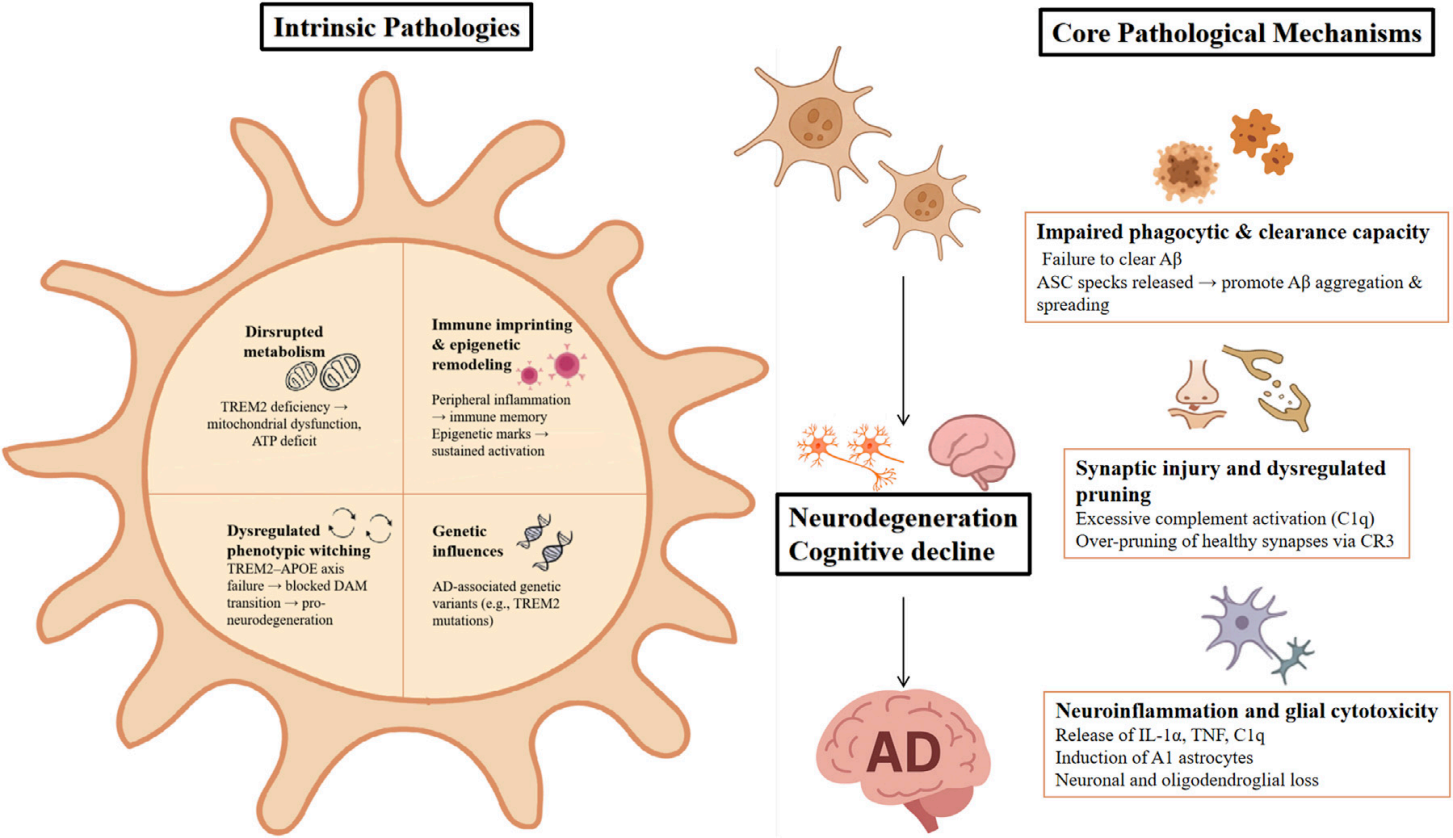
Acknowledged Findings on Microglial Dysfunction in AD. Intrinsic microglial alterations in AD include [1] disrupted metabolism (TREM2 deficiency, mitochondrial dysfunction) [2], impaired phenotypic switching (TREM2–APOE axis failure, blocked DAM transition) [3], immune imprinting and epigenetic remodeling (peripheral inflammation, maladaptive epigenetic marks), and [4] genetic influences (TREM2 mutations). These abnormalities drive [1] impaired Aβ clearance (defective phagocytosis, ASC speck–mediated aggregation) [2], synaptic injury and dysregulated pruning (C1q overactivation, CR3-dependent loss of synapses), and [3] neuroinflammation with glial cytotoxicity (release of IL-1α, TNF, C1q; induction of A1 astrocytes; neuronal and oligodendroglial loss). Final outcome involves neurodegeneration and cognitive decline in AD. Abbreviations: AD, Alzheimer’s disease; DAM, disease-associated microglia; Aβ, amyloid-β; APOE, apolipoprotein E; CR3, complement receptor 3; IL, interleukin; TNF, tumor necrosis factor.

In conclusion, microglial dysfunction contributes to Aβ accumulation, synaptic degeneration, neuroinflammation, and neuronal loss in AD. These functional and phenotypic impairments are modulated by both genetic and epigenetic factors, as shown in Figure 6. Altogether, these alterations constitute the neuroimmune foundation of AD and underscore microglia as a promising target for therapeutic intervention.

### Research hotspots

Analysis of articles from the WoSCC database over the past decade provides insights into research hotspots on microglia in AD. The progression of burst keywords across time highlights transitions of research hotspots in this field. During 2015–2022, burst keywords such as “*in vivo*,” “amyloid precursor protein,”“*in vitro*,”“central nervous system,”“oxidative stress,”“reveals,“and “TREM2 deficiency” suggested a predominant emphasis on molecular pathology, neurodegeneration, and associated gene defects in early research. In particular, “amyloid precursor protein” is a pivotal molecule implicated in AD, with its aberrant processing leading to β-amyloid (Aβ) deposition and neuronal injury [32]. Integrating “*in vivo*” and “*in vitro*” approaches has offered crucial strategies for uncovering AD’s molecular underpinnings [33, 34]. The degenerative changes in the “central nervous system” have emerged as a core focus, especially concerning microglial mechanisms in AD pathogenesis [35, 36]. “Oxidative stress” alters microglial activity and contributes to the aggravation of neurodegeneration in AD [37]. The keyword “reveals” is commonly used in research findings to highlight the validated neurotoxicity of specific molecules (e.g., Aβ) or their efficacy as therapeutic targets [38]. “TREM2 deficiency” contributes significantly to microglial impairment, with TREM2 being essential for phagocytic activity, inflammatory modulation, and homeostatic balance. Moreover, mutations in this gene compromise these functions, promoting Aβ deposition and tau propagation [39, 40].

The keywords persisting until 2024 reflect current research hotspots: “memory,” “NLRP3 inflammasome,” and “system.”

“Memory”, as a core component of cognitive function, deteriorates characteristically in neurodegenerative diseases such as AD [41]. Evidence indicates that memory impairment in AD patients is associated not only with Aβ accumulation and tau pathology but also directly with synaptic plasticity deficits caused by microglial dysfunction [42, 43]. Moreover, hippocampal atrophy and functional decline observed in AD are linked to neuroinflammation and synaptic damage driven by abnormal microglial activity [44, 45]. For instance, Li et al. demonstrated that hippocampal microglia regulate cellular communication through CCL and CSF signaling crosstalk, contributing to AD-related cognitive and memory decline [46]. Similarly, Wei Lei et al. showed that increased H3K18 lactylation in aging microglia activates the hippocampal NF-κB pathway, promoting inflammation and exacerbating AD-associated cognitive deficits [47]. Ye et al. observed increased AIM2 levels in microglia of AD mice, and targeted knockout of AIM2 in microglia markedly ameliorates synaptic plasticity disruptions and spatial memory deficits in these mice [48]. These studies suggest that memory impairments in AD patients are closely linked to microglia-driven neuropathological alterations, synaptic dysfunction, and cascades of neuroinflammation. Through modulating neuroinflammation, synaptic plasticity, and multiple other pathways, microglia are crucial contributors to the development and progression of memory impairments in AD.

“NLRP3 inflammasome”, an essential element of immune responses, exerts a prominent role in neuroinflammation driven by microglia. In AD, microglia sense pathogens or danger signals through receptors like TLRs, which trigger activation of NF-κB and the NLRP3 inflammasome. This inflammasome activates caspase-1, cleaves GSDMD to induce pyroptosis, and releases IL-1β and IL-18, further facilitating Aβ plaque propagation, which amplifies inflammation and neuronal injury. The death of neurons activates microglia, forming a vicious feedback loop that accelerates neurodegeneration [49, 50]. According to Zhang et al., abnormal glutamine metabolism in microglia under AD pathology suppresses mitophagy through the AMPK/mTORC1 signaling pathway, resulting in reactive oxygen species (ROS) accumulation and selective activation of the NLRP3 inflammasome. Moreover, inhibition of glutaminase can block this process and improve cognitive impairment in AD [51]. Moonen et al. found distinct cell type-specific patterns of pyroptosis activation in AD brains: microglia showed classical activation of the NLRP3-ASC-caspase-1 pathway, while astrocytes and neurons induced GSDMD cleavage through caspase-8 and caspase-4, respectively [52]. These discoveries reveal that the microglial NLRP3 inflammasome contributes to neurodegeneration via distinct molecular pathways, offering new molecular targets for therapeutic strategies.

The keyword “system” denotes microglia as essential immune cells within the central nervous system, involved in AD progression through multiple critical systems. At the immune system level, Juul-Madsen et al. expanded the understanding of the peripheral-central immune system. Notably, he revealed that the complement receptor system mediates selective phagocytosis and lysosomal clearance of Aβ by peripheral monocytes and central microglia. Consequently, dysfunction in this system could be a key hallmark of the AD prodromal phase [53]. At the metabolic regulation level, Kaji et al. found that defective handling of APOE protein by microglia serves as a trigger for AD pathology. This lysosomal impairment promotes APOE protofibril buildup, which subsequently activates the JAK/STAT signaling pathway, leading to Aβ amyloid formation and plaque deposition, contributing to AD progression [54]. Haney et al. reported that the AD high-risk genotype APOE4/4 promotes lipid droplet accumulation in microglia via ACSL1 mediation, leading to Tau phosphorylation and neurotoxicity, highlighting lipid metabolic dysregulation as a key mechanism in AD pathogenesis [55]. At the cellular heterogeneity level, Wu et al. applied single-cell sequencing and identified 11 distinct microglial subtypes. Among these, AD-associated subpopulations exhibited significant synaptic dysfunction, revealing the intricate molecular heterogeneity of microglial responses in AD and providing a novel framework for targeted diagnosis and treatment [56]. Moreover, advances in technological intervention systems. Feng et al. developed a CNP-cardiolipin nanosystem that modulates the TLR4/NF-κB pathway to induce microglial polarization toward the M2 phenotype, thereby effectively improving AD pathology [13].

### Trends discussion

The growing number of articles on microglia in AD highlights the prominence of this field, with recent emerging burst keywords and high-impact articles signaling shifting trends. This suggests that research focusing on microglia as a breakthrough point for neurodegenerative diseases is likely to become a major future direction. The keywords persisting until 2024- “memory,” “NLRP3 inflammasome,” and “system”- represent current research hotspots. Future research is expected to further focus on the molecular mechanisms by which microglia regulate neuroinflammation and synaptic plasticity, with particular attention to programmed cell death (such as pyroptosis) mediated by the NLRP3 inflammasome signaling pathway in the pathogenesis of AD. Meanwhile, with the advancement of systems biology, increasing emphasis will be placed on the multi-level interactions of microglia within immune, metabolic, and neural systems, elucidating their pivotal roles in systemic dysregulation associated with neurodegenerative diseases. High-impact studies have already employed cutting-edge technologies such as single-cell transcriptomics, genetic analysis, and single-nucleus RNA sequencing to uncover microglial heterogeneity and their multidimensional roles in AD pathology. These advanced techniques are expected to provide essential methodological support for future research, facilitating deeper exploration of microglial regulatory networks and their potential as therapeutic targets in AD. Overall, future investigations are likely to further elucidate the system-level functions of microglia in neurodegenerative diseases, offering new perspectives for early diagnosis and precision intervention.

### Limitations

This study has several limitations that may compromise the comprehensiveness and generalizability of the findings. First, to ensure literature quality, only the WoSCC database was used, which might have led to an incomplete retrieval of relevant studies. Employing multiple databases could offer a broader perspective. Second, the search was restricted to English-language articles, potentially excluding valuable research published in other languages. Third, the document type was limited to article, possibly overlooking important contributions from other formats. Due to time constraints and the focus on emerging hotspots and trends, only articles from the past 10 years were included, which may have resulted in partial coverage of the field. Furthermore, some relevant research findings may not be publicly available in the published literature.

Moreover, the research content of the selected articles presents several limitations. Current studies on the role of microglia in AD largely remain at the basic research stage. Methodologically, most existing studies rely on *in vivo* experiments using animal models (such as transgenic AD mice) and *in vitro* cell line studies, while direct evidence from human subjects remains limited. Although some investigations have examined human tissue samples, these efforts are constrained by small sample sizes and incomplete clinical information. In terms of mechanistic research, several pathological processes have been identified, including NLRP3 inflammasome activation, metabolic reprogramming, and synaptic pruning abnormalities. However, the lack of systematic integration and the methodological heterogeneity across laboratories have resulted in fragmented and sometimes inconsistent findings. Furthermore, most therapeutic interventions targeting microglia are still in the experimental or early clinical research phases, facing technical challenges such as poor blood-brain barrier permeability, limited specificity, and potential adverse effects. Given the substantial heterogeneity of microglia and the individual variability among AD patients, achieving precise and personalized interventions.

## Conclusion

This study systematically analyzed microglia-related research in AD over the past decade through bibliometric methods, offering a comprehensive overview of the field’s current landscape, emerging hotspots, and future trends, while providing an in-depth analysis of the underlying mechanisms. The results indicate a growing global interest in the mechanisms of microglial involvement in AD since 2021, with the United States and China emerging as the most influential contributors. Leading institutions include the University of California system and Harvard University. *Journal of Neuroinflammation* was the journal with the highest number of articles. Through NLRP3 inflammasome activation, microglia orchestrate neuroinflammatory responses in concert with systemic immune, metabolic, and cellular alterations, contributing to AD’s pathology and cognitive deficits—a rapidly expanding area of research interest. High-impact articles concentrate on microglial activation, metabolic regulation, epigenetic alterations, inflammasome signaling, and genetic variants, offering theoretical insights into disease pathogenesis and guiding therapeutic development. However, an in-depth investigation into the dynamic transitions and regulatory networks of microglia across different AD stages is essential to support early diagnosis and precision therapy, ultimately improving clinical outcomes.

## Data Availability

The original contributions presented in the study are included in the article/supplementary material, further inquiries can be directed to the corresponding authors.

## References

[1] Scheltens P De Strooper B Kivipelto M Holstege H Chételat G Teunissen CE Alzheimer’s disease. The Lancet (2021) 397(10284):1577–1590. 10.1016/S0140-6736(20)32205-4 33667416 PMC8354300

[2] Twarowski B Herbet M . Inflammatory processes in Alzheimer’s disease-pathomechanism, diagnosis and treatment: a review. Int J Mol Sci (2023) 24(7):6518. 10.3390/ijms24076518 37047492 PMC10095343

[3] Yeung CHC Schooling CM . Systemic inflammatory regulators and risk of Alzheimer’s disease: a bidirectional mendelian-randomization study. Int J Epidemiol (2021) 50(3):829–840. 10.1093/ije/dyaa241 33313759

[4] Wang S Mustafa M Yuede CM Salazar SV Kong P Long H Anti-human TREM2 induces microglia proliferation and reduces pathology in an Alzheimer’s disease model. J Exp Med (2020) 217(9):e20200785. 10.1084/jem.20200785 32579671 PMC7478730

[5] Sun N Victor MB Park YP Xiong X Scannail AN Leary N Human microglial state dynamics in Alzheimer’s disease progression. Cell (2023) 186(20):4386–403.e29. 10.1016/j.cell.2023.08.037 37774678 PMC10644954

[6] Zhao X Sun J Xiong L She L Li L Tang H β-amyloid binds to microglia Dectin-1 to induce inflammatory response in the pathogenesis of Alzheimer’s disease. Int J Biol Sci (2023) 19(10):3249–65. 10.7150/ijbs.81900 37416769 PMC10321287

[7] Liu PP Liu XH Ren MJ Liu XT Shi XQ Li ML Neuronal cathepsin S increases neuroinflammation and causes cognitive decline via CX3CL1-CX3CR1 axis and JAK2-STAT3 pathway in aging and Alzheimer’s disease. Aging Cell (2025) 24(2):e14393. 10.1111/acel.14393 39453382 PMC11822647

[8] Zuppe H Reed E . Common cytokine receptor gamma chain family cytokines activate MAPK, PI3K, and JAK/STAT pathways in microglia to influence Alzheimer’s disease. Front Mol Neurosci (2024) 17:1441691. 10.3389/fnmol.2024.1441691 39324116 PMC11422389

[9] Cangalaya C Wegmann S Sun W Diez L Gottfried A Richter K Real-time mechanisms of exacerbated synaptic remodeling by microglia in acute models of systemic inflammation and tauopathy. Brain Behav Immun (2023) 110:245–259. 10.1016/j.bbi.2023.02.023 36906076

[10] Lan G Chen X Yang J Sun P Cai Y Li A Microglial reactivity correlates with presynaptic loss independent of β-Amyloid and tau. Ann Neurol (2024) 95(5):917–928. 10.1002/ana.26885 38356322 PMC11060909

[11] Schlepckow K Morenas-Rodríguez E Hong S Haass C . Stimulation of TREM2 with agonistic antibodies-an emerging therapeutic option for Alzheimer’s disease. Lancet Neurol (2023) 22(11):1048–60. 10.1016/S1474-4422(23)00247-8 37863592

[12] Long H Simmons A Mayorga A Burgess B Nguyen T Budda B Preclinical and first-in-human evaluation of AL002, a novel TREM2 agonistic antibody for Alzheimer’s disease. Alzheimer's Res and Ther (2024) 16(1):235. 10.1186/s13195-024-01599-1 39444037 PMC11515656

[13] Feng Q Zhang X Zhao X Liu J Wang Q Yao Y Intranasal delivery of pure nanodrug loaded liposomes for Alzheimer’s disease treatment by efficiently regulating microglial polarization. Small (2024) 20(50):e2405781. 10.1002/smll.202405781 39370581

[14] Sanjay SJH Park M Lee HJ . Cyanidin-3-O-Glucoside regulates the M1/M2 polarization of microglia via PPARγ and Aβ42 phagocytosis through TREM2 in an alzheimer’s disease model. Mol Neurobiol (2022) 61(2):1223. 10.1007/s12035-023-03877-9 PMC10861610 38129702

[15] Raikwar SP Thangavel R Dubova I Selvakumar GP Ahmed ME Kempuraj D Targeted gene editing of glia maturation factor in microglia: a novel Alzheimer’s disease therapeutic target. Mol Neurobiol (2019) 56(1):378–93. 10.1007/s12035-018-1068-y 29704201 PMC6344368

[16] Meier S Larsen ASG Wanke F Mercado N Mei A Takacs L An efficient, non-viral arrayed CRISPR screening platform for iPSC-derived myeloid and microglia models. Stem Cell Rep (2025) 20(3):102420. 10.1016/j.stemcr.2025.102420 39983727 PMC11960525

[17] Guo M Gong D Yang W . In-depth analysis of research hotspots and emerging trends in AI for retinal diseases over the past decade. Front Med (2024) 11:1489139. 10.3389/fmed.2024.1489139 39635592 PMC11614663

[18] Jia Q Wang X Li X Xie C Zhang Q Mu J Analysis of research hotspots and trends in pediatric ophthalmopathy based on 10 years of WoSCC literature. Front Pediatr (2024) 12:1405110. 10.3389/fped.2024.1405110 38873588 PMC11171143

[19] Xie J Van Hoecke L Vandenbroucke RE . The impact of systemic inflammation on Alzheimer’s disease pathology. Front Immunol (2022) 12:796867. 10.3389/fimmu.2021.796867 35069578 PMC8770958

[20] Guerrero A De Strooper B Arancibia-Cárcamo IL . Cellular senescence at the crossroads of inflammation and Alzheimer’s disease. Trends Neurosci (2021) 44(9):714–27. 10.1016/j.tins.2021.06.007 34366147

[21] Baligács N Albertini G Borrie SC Serneels L Pridans C Balusu S Homeostatic microglia initially seed and activated microglia later reshape amyloid plaques in Alzheimer’s disease. Nat Commun (2024) 15(1):10634. 10.1038/s41467-024-54779-w 39639016 PMC11621353

[22] Yeh FL Wang Y Tom I Gonzalez LC Sheng M . TREM2 binds to apolipoproteins, including APOE and CLU/APOJ, and thereby facilitates uptake of amyloid-beta by microglia. Neuron (2016) 91(2):328–40. 10.1016/j.neuron.2016.06.015 27477018

[23] Venegas C Kumar S Franklin BS Dierkes T Brinkschulte R Tejera D Microglia-derived ASC specks cross-seed amyloid-β in Alzheimer’s disease. Nature (2017) 552(7685):355–61. 10.1038/nature25158 29293211

[24] Hong S Beja-Glasser VF Nfonoyim BM Frouin A Li S Ramakrishnan S Complement and microglia mediate early synapse loss in Alzheimer mouse models. Science (2016) 352(6286):712–6. 10.1126/science.aad8373 27033548 PMC5094372

[25] Liddelow SA Guttenplan KA Clarke LE Bennett FC Bohlen CJ Schirmer L Neurotoxic reactive astrocytes are induced by activated microglia. Nature (2017) 541(7638):481–7. 10.1038/nature21029 28099414 PMC5404890

[26] Ulland TK Song WM Huang SC Ulrich JD Sergushichev A Beatty WL TREM2 maintains microglial metabolic fitness in Alzheimer’s disease. Cell (2017) 170(4):649–63.e13. 10.1016/j.cell.2017.07.023 28802038 PMC5573224

[27] Keren-Shaul H Spinrad A Weiner A Matcovitch-Natan O Dvir-Szternfeld R Ulland TK A unique microglia type associated with restricting development of Alzheimer’s disease. Cell (2017) 169(7):1276–90.e17. 10.1016/j.cell.2017.05.018 28602351

[28] Krasemann S Madore C Cialic R Baufeld C Calcagno N El Fatimy R The TREM2-APOE pathway drives the transcriptional phenotype of dysfunctional microglia in neurodegenerative diseases. Immunity (2017) 47(3):566–81.e9. 10.1016/j.immuni.2017.08.008 28930663 PMC5719893

[29] Wendeln AC Degenhardt K Kaurani L Gertig M Ulas T Jain G Innate immune memory in the brain shapes neurological disease hallmarks. Nature (2018) 556(7701):332–8. 10.1038/s41586-018-0023-4 29643512 PMC6038912

[30] Sims R van der Lee SJ Naj AC Bellenguez C Badarinarayan N Jakobsdottir J Rare coding variants in PLCG2, ABI3, and TREM2 implicate microglial-mediated innate immunity in Alzheimer’s disease. Nat Genet (2017) 49(9):1373–84. 10.1038/ng.3916 28714976 PMC5669039

[31] Zhou Y Song WM Andhey PS Swain A Levy T Miller KR Human and mouse single-nucleus transcriptomics reveal TREM2-dependent and TREM2-independent cellular responses in Alzheimer’s disease. Nat Med (2020) 26(1):131–42. 10.1038/s41591-019-0695-9 31932797 PMC6980793

[32] Gerrits E Brouwer N Kooistra SM Woodbury ME Vermeiren Y Lambourne M Distinct amyloid-β and tau-associated microglia profiles in Alzheimer’s disease. Acta Neuropathol (2021) 141(5):681–96. 10.1007/s00401-021-02263-w 33609158 PMC8043951

[33] Zhu M Liu Y Chen C Chen H Ni W Song Y TLR4/Rac1/NLRP3 pathway mediates Amyloid-β-Induced neuroinflammation in Alzheimer’s disease. J Alzheimer’s Dis (2024) 99(3):911–925. 10.3233/JAD-240012 38728187

[34] Wang D Liu J Zhu Q Wei X Zhang X Chen Q Ouabain ameliorates Alzheimer’s disease-associated neuropathology and cognitive impairment in FAD4T mice. Nutrients (2024) 16(20):3558. 10.3390/nu16203558 39458551 PMC11510559

[35] Morris GP Foster CG Courtney JM Collins JM Cashion JM Brown LS Microglia directly associate with pericytes in the central nervous system. Glia (2023) 71(8):1847–69. 10.1002/glia.24371 36994950 PMC10952742

[36] Chen H Guo Z Sun Y Dai X . The immunometabolic reprogramming of microglia in Alzheimer’s disease. Neurochem Int (2023) 171:105614. 10.1016/j.neuint.2023.105614 37748710

[37] Kang YJ Hyeon SJ McQuade A Lim J Baek SH Diep YN Neurotoxic microglial activation via IFNγ-Induced Nrf2 reduction exacerbating Alzheimer’s disease. Adv Sci (Weinh) (2024) 11(20):e2304357. 10.1002/advs.202304357 PMC11132036 38482922

[38] Xia D Lianoglou S Sandmann T Calvert M Suh JH Thomsen E Novel app knock-in mouse model shows key features of amyloid pathology and reveals profound metabolic dysregulation of microglia. Mol Neurodegeneration (2022) 17(1):41. 10.1186/s13024-022-00547-7 35690868 PMC9188195

[39] Lee-Gosselin A Jury-Garfe N You Y Dabin L Soni D Dutta S TREM2-Deficient microglia attenuate Tau spreading *in vivo* . Cells (2023) 12(12):1597. 10.3390/cells12121597 37371067 PMC10296847

[40] Stoiljkovic M Gutierrez KO Kelley C Horvath TL Hajós M . TREM2 deficiency disrupts network oscillations leading to epileptic activity and aggravates Amyloid-β-Related hippocampal pathophysiology in mice. J Alzheimer's Dis (2022) 88(3):837–847. 10.3233/JAD-210041 34120899 PMC8898080

[41] Kamatham PT Shukla R Khatri DK Vora LK . Pathogenesis, diagnostics, and therapeutics for Alzheimer’s disease: breaking the memory barrier. Ageing Res Rev (2024) 101:102481. 10.1016/j.arr.2024.102481 39236855

[42] Wang W Li Y Ma F Sheng X Chen K Zhuo R Microglial repopulation reverses cognitive and synaptic deficits in an Alzheimer’s disease model by restoring BDNF signaling. Brain Behav Immun (2023) 113:275–288. 10.1016/j.bbi.2023.07.011 37482204

[43] Udeochu JC Amin S Huang Y Fan L Torres ERS Carling GK Tau activation of microglial cGAS-IFN reduces MEF2C-mediated cognitive resilience. Nat Neurosci (2023) 26(5):737–750. 10.1038/s41593-023-01315-6 37095396 PMC10166855

[44] Falcicchia C Tozzi F Gabrielli M Amoretti S Masini G Nardi G Microglial extracellular vesicles induce Alzheimer’s disease-related cortico-hippocampal network dysfunction. Brain Commun (2023) 5(3):fcad170. 10.1093/braincomms/fcad170 37288314 PMC10243901

[45] Rao YL Ganaraja B Murlimanju BV Joy T Krishnamurthy A Agrawal A . Hippocampus and its involvement in Alzheimer’s disease: a review. 3 Biotech (2022) 12(2):55. 10.1007/s13205-022-03123-4 35116217 PMC8807768

[46] Li H Ye T Liu X Guo R Yang X Li Y The role of signaling crosstalk of microglia in hippocampus on progression of ageing and Alzheimer’s disease. J Pharm Anal (2023) 13(7):788–805. 10.1016/j.jpha.2023.05.008 37577391 PMC10422165

[47] Wei L Yang X Wang J Wang Z Wang Q Ding Y H3K18 lactylation of senescent microglia potentiates brain aging and Alzheimer’s disease through the NFκB signaling pathway. J Neuroinflammation (2023) 20(1):208. 10.1186/s12974-023-02879-7 37697347 PMC10494370

[48] Ye L Hu M Mao R Tan Y Sun M Jia J Conditional knockout of AIM2 in microglia ameliorates synaptic plasticity and spatial memory deficits in a mouse model of Alzheimer’s disease. CNS Neurosci Ther (2024) 30(6):e14555. 10.1111/cns.14555 38105588 PMC11163192

[49] Liu Y Dai Y Li Q Chen C Chen H Song Y Beta-amyloid activates NLRP3 inflammasome via TLR4 in mouse microglia. Neurosci Lett (2020) 736:135279. 10.1016/j.neulet.2020.135279 32726591

[50] Ayyubova G Madhu LN . Microglial NLRP3 inflammasomes in Alzheimer’s disease pathogenesis: from interaction with autophagy/mitophagy to therapeutics. Mol Neurobiol (2025) 62(6):7124–43. 10.1007/s12035-025-04758-z 39951189

[51] Zhang Z Li M Li X Feng Z Luo G Wang Y Glutamine metabolism modulates microglial NLRP3 inflammasome activity through mitophagy in Alzheimer’s disease. J Neuroinflammation (2024) 21(1):261. 10.1186/s12974-024-03254-w 39407211 PMC11481753

[52] Moonen S Koper MJ Van Schoor E Schaeverbeke JM Vandenberghe R von Arnim CAF Pyroptosis in Alzheimer’s disease: cell type-specific activation in microglia, astrocytes and neurons. Acta Neuropathol (2023) 145(2):175–95. 10.1007/s00401-022-02528-y 36481964

[53] Juul-Madsen K Parbo P Ismail R Ovesen PL Schmidt V Madsen LS Amyloid-β aggregates activate peripheral monocytes in mild cognitive impairment. Nat Commun (2024) 15(1):1224. 10.1038/s41467-024-45627-y 38336934 PMC10858199

[54] Kaji S Berghoff SA Spieth L Schlaphoff L Sasmita AO Vitale S Apolipoprotein E aggregation in microglia initiates Alzheimer’s disease pathology by seeding β-amyloidosis. Immunity (2024) 57(11):2651–68.e12. 10.1016/j.immuni.2024.09.014 39419029

[55] Haney MS Pálovics R Munson CN Long C Johansson PK Yip O APOE4/4 is linked to damaging lipid droplets in Alzheimer’s disease microglia. Nature (2024) 628(8006):154–161. 10.1038/s41586-024-07185-7 38480892 PMC10990924

[56] Wu X Liu M Zhang X Pan X Cui X Jin J Elucidating microglial heterogeneity and functions in Alzheimer’s disease using single-cell analysis and convolutional neural network disease model construction. Sci Rep (2024) 14(1):17271. 10.1038/s41598-024-67537-1 39068182 PMC11283484

